# Enzymes@ZIF-8 Nanocomposites with Protection Nanocoating: Stability and Acid-Resistant Evaluation

**DOI:** 10.3390/polym11010027

**Published:** 2018-12-25

**Authors:** Yuxiao Feng, Le Zhong, Muhammad Bilal, Zhilei Tan, Ying Hou, Shiru Jia, Jiandong Cui

**Affiliations:** 1 State Key Laboratory of Food Nutrition and Safety, Key Laboratory of Industrial Fermentation Microbiology, Ministry of Education, Tianjin University of Science and Technology, No 29, 13th, Avenue, Tianjin Economic and Technological Development Area (TEDA), Tianjin 300457, China; cjd007cn@163.com (Y.F.); 13230470931@126.com (L.Z.); tanzhilei@tust.edu.cn (Z.T.); houying@tust.edu.cn (Y.H.); 2 School of Life Science and Food Engineering, Huaiyin Institute of Technology, Huaian 223003, China; bilaluaf@sjtu.edu.cn

**Keywords:** enzyme/ZIF-8 nanocomposites, nanocoating, stability, acid resistance

## Abstract

Zeolitic imidazole framework-8 (ZIF-8) with tunable pore sizes and high surface areas have recently emerged as a promising support for immobilizing enzymes. However, the instability in the aqueous acidic environment and difficulty of recovery has limited their practical applications in some cases. In this study, catalase/ZIF-8 composites with a protective nanocoating were prepared by the controlled self-assembly of silanes or coordination complexes (tannic acid (TA) and Fe^3+^). The properties of the catalase (CAT)/ZIF-8 composites with a protective nanocoating were also determined. The recovered activity of CAT/ZIF-8 and CAT/ZIF-8 with protective nanocoating was 70% and 65%, respectively. Compared with the conventional CAT/ZIF-8 composites, CAT/ZIF-8 with protective nanocoating exhibited excellent acid resistance. For example, after treatment for 60 min in phosphate buffer solution (pH 3.0), CAT/ZIF-8 composites only maintained 20% of their initial activity (about 12 U/mg). However, CAT/ZIF-8 with a protective nanocoating could still retain about 50% of its initial activity (about 10 U/mg). Meanwhile, the thermostability and storage stability of the CAT/ZIF-8 composites was enhanced significantly due to the presence of nanocoating compared with conventional CAT/ZIF-8. More importantly, the CAT/ZIF-8 with a protective nanocoating retained 40% of its initial activity after 7 cycles, whereas CAT/ZIF-8 only retained 8% of the initial activity. The approach in this study could be an efficient strategy to prepare enzyme/ZIF-8 composites with both high acid resistance and excellent recyclability.

## 1. Introduction

Enzymes, as natural catalysts, have long been of abundant interest for the fine chemicals, pharmaceutical, cosmetic, and food industries [1,2]. However, their low stability, and the difficulty in the recovery and reusability of enzymes hampers their implementation in industries [3,4]. In this regard, immobilization can overcome these disadvantages [5,6]. Generally, immobilization technology is able to prevent subunit dissociation, aggregation, and autolysis or proteolysis. Multipoint covalent immobilization can also increase the rigidification of the enzyme and produce better microenvironments [7,8]. Furthermore, the solid supports for enzyme immobilization include microparticles [9], silica gel [10], hydrogels [11], and nanoporous materials [12]. However, these conventional supports for enzymes are not finely tunable and crystalline, thus exhibiting non-uniformity and long-range ordering from the atomic to microscale regime, which leads to low protein-loading efficiency, low stability, and/or enzyme leaching [13,14,15]. Therefore, it is very important to find an immobilization carrier and relative technique that cause the minimum loss of biocatalytic activity.

**Figure 1:**
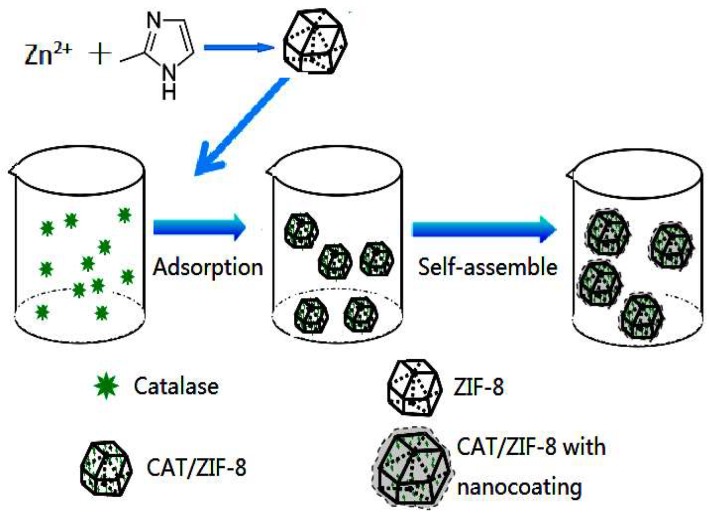
Schematic illustration of the synthesis of catalase (CAT)/ Zeolitic imidazole framework (ZIF)-8, Fe^3+^-TA@CAT/ZIF-8, and SiO_2_@CAT@ZIF-8.

In recent years, a new interesting class of hybrid porous material, called the metal–organic framework (MOF), has generated a great deal of research interest. MOFs are a new type of porous hybrid materials and are constructed by metal ions and organic ligands which are linked together by strong coordination bonds [16,17,18]. Owing to their well-defined pore structures with high surface areas and chemical functionality, MOFs serve as promising candidates for the immobilization of enzymes [19,20,21]. Four main routes have been described for the immobilization of enzymes into/onto MOFs: surface adsorption, covalent linkage, diffusion into the pores of MOF, and co-precipitation [19,22]. Among these routes, the surface adsorption has the widest generality, as any stable MOFs can be employed without the consideration of the dimensions of enzyme molecules. For surface immobilization, enzymes are usually immobilized on MOFs by a relatively weak physical interaction such as hydrogen bonds, ionic bands, molecular interaction and so on [23]. Among the different kinds of MOFs, zeolitic imidazole frameworks-8 (ZIF-8) is widely known and is often used for the immobilization of enzymes because of their high surface area, exceptional chemical and thermal stability, and ease of synthesis in alcohol or water phase [24,25]. However, enzymes adsorbed on the surface of ZIF-8 may suffer significant leaching during recycled usage due to the weak non-covalent interactions between enzymes and ZIF-8. Furthermore, the enzymes adsorbed on the external surface of ZIF-8 particles were directly exposed in the external environment and suffer inactivation caused by denaturing stresses and a hazardous external environment [8]. Therefore, the stability issue should be paid special attention when the surface immobilization strategy is adopted for enzyme immobilization. Especially, the structure of ZIF-8 crystals would be easily broken under acidic conditions, which would obstruct the extension of the applied range of hybrid composites under acidic conditions [26,27]. Overcoming these limitations would facilitate the fabrication of novel enzymes@MOF composites and their exploitation for bioapplications [28]. Catalase (CAT) is a multimeric enzyme and is applied in various fields including the food industry and for environmental protection. However, CAT is easy to inactivate due to its subunit dissociation [29,30]. Furthermore, as a substrate of CAT, hydrogen peroxide is able to chemically modify the peptide core of CAT, and also produce the oxidation of some cofactors and prosthetic groups [31]. To overcome these problems, CAT was immobilized onto/into various supports, such as activated glyoxyl or glutaraldehyde agarose [32], functionalized polymer [33], and mesoporous silica sphere [34]. Moreover, CAT was successfully embedded into the uniformly sized ZIFs crystals, and the resultant CAT/ZIFs composites exhibited high activity [35,36]. However, the CAT/ZIFs composites are extremely unstable in the aqueous acidic environment and easily decomposed by acids, thus causing enzyme leakage and inactivation under acid conditions. In this study, CAT was adsorbed on the surface of ZIF-8, and then CAT/ZIF-8 composites with a protective nanocoating were prepared by the controlled self-assembly of silanes or coordination complexes (tannic acid (TA) and Fe^3+^). The preparation process consists of a sequential reaction involving the adsorption of CAT on the surface of prefabricated ZIF-8, the controlled self-assembly of silanes, or Fe^3+^-TA coordination complexes, and thus results in the growth of a mesoporous nanocoating (silica layer or Fe^3+^-CA film) on the surface of the immobilized CAT on ZIF-8 particles (Figure 1). The CAT/ZIF-8 composites with a protective nanocoating showed excellent recyclability and stability against denaturants and heat. More interestingly, we found that the stability of CAT/ZIF-8 composites with a protective nanocoating was significantly improved in low pH solution, which might greatly extend the enzymes/MOF composite application range in future.

## 2. Materials and Methods

### 2.1. Materials

Fluorescein isothiocyanate (FITC), 2-methylimidazole, tannic acid, hydrogen peroxide (H_2_O_2_, 30%), tetramethoxysilane, and zinc nitrate were purchased from International Aladdin Reagent Inc. (Shanghai, China). Catalase (EC 1.11.1.6 from bovine liver, 1 × 10^4^ U/mg protein) was purchased from Sigma-Aldrich (Shanghai, China). Tris, Bradford reagent, coomassie brilliant blue R-250 were from Beijing Chemical Reagent Company (Beijing, China).

### 2.2. Synthesis of the ZIF-8 and Catalase/ZIF-8 Composites (CAT/ZIF-8)

For the ZIF-8, 2-methylimidazole water solution (0.05 M, 10 mL) was added into Zn(NO_3_)_2_ water solution (0.3 M, 1 mL). After the mixture was stirred for 30 min, the product was collected by centrifuging at 6000× *g* for 10 min, washed with deionized water three times and dried. For CAT/ZIF-8, a certain amount of the ZIF-8 (0.1 g) was immersed in 1 mL of catalase solution (1 mg/mL) for 3 h with stirring at 25 °C. After centrifugation at 6000× *g* for 10 min, the resulting precipitate was washed with DI water three times and lyophilized. 

### 2.3. Synthesis of Catalase/ZIF-8 Composites with a Protective Nanocoating

For the catalase/ZIF-8 composites with mesoporous silica shell (SiO_2_@CAT/ZIF-8), 1.2 mL cetyltrimethylammonium bromide (CTAB) (0.05%) and 75 μL tetramethoxysilane (TMOS) (98%) were added to the aqueous CAT/ZIF-8 suspension. The suspension was vigorously mixed for 1 h. The product SiO_2_CAT/ZIFs was recovered by centrifugation at 10,000× *g* for 10 min and washed by DI water three times, and re-suspended in DI water. For catalase/ZIF-8 composites with Fe^3+^-TA film (Fe^3+^-TA@CAT/ZIF-8), 5 µL FeCl_3_·6H_2_O (10 mg/mL) and TA (40 mg/mL) solutions were added to the aqueous CAT/ZIF-8 suspension (500 µL). The suspension was mixed for 10 min. The product Fe^3+^-TA@CAT/ZIF-8 was separated by centrifugation, washed by DI water, and re-suspended in DI water.

### 2.4. Characterization Methods

Scanning electron microscopy (SEM) was performed by JEOL JSM6700 (Japan electron optics laboratory CO., LTD, Beijing, China), and the acceleration voltage was 15 kV. Transmission electron microscope (TEM) images were obtained on JEOL JEM2100 (Japan electron optics laboratory CO., LTD, Beijing, China) operated at 120 kV. Fourier transform infrared (FTIR) spectra were obtained using a NEXUS870 infrared spectrometer (Thermo Nicolet Corporation, Madison, WI, USA) using the standard KBr disk method. FT-IR measurements were conducted in the region of 400–4000 cm^−1^. Powder X-ray diffraction (PXRD) patterns were recorded using a X-ray powder diffraction (D/Max-2500 diffractometer, Shimadzu, Japan) at 40 kV and 40 mA. The elemental composition was obtained by using an energy-dispersive spectrometer (EDS) (S2 Ranger, Bruker, Karlsruhe, Germany).

### 2.5. Labeled Catalase with FITC

Catalase (50 mg) was mixed with FITC solution (50 mg/mL, FITC in acetone) for 3 min. Modified FITC-labeled catalase was then absorbed on ZIF-8. Confocal laser scanning microscopy (CLSM) observation was performed with a Leica TCS SP5 microscope (Leica Camera AG, Wetzlar, Germany). The samples were excited at 390 nm, and FITC fluorescence was detected between 460 and 480 nm.

### 2.6. Activity Assay

The activities of free catalase and immobilized catalase were measured by the modification of the procedure in [37]. A small amount of enzyme samples were added to phosphate buffer solution (pH 7.0, 50 mM) and then incubated at 30 °C for 1 h. After incubation, the samples were added to 0.2% hydrogen peroxide solution, and the final concentrations of hydrogen peroxide were monitored by measuring their absorbance at 240 nm on a 2800H spectrophotometer (Unicoi Instrument Co., Ltd. Shanghai, China). One unit of catalase activity is defined as the amount of enzyme that decomposes 1 μmol of hydrogen peroxide per minute.

### 2.7. Acid Tolerance Measurement (pH 3.0) of SiO_2_@CAT/ZIF-8 and Fe^3+^-CA@CAT/ZIF-8

The CAT/ZIF-8, SiO_2_@CAT/ZIF-8 and Fe^3+^-TA@CAT/ZIF-8 were added into 2.5 mL phosphate buffer solution (pH 3.0) and incubated for 30–90 min. After incubation, the morphological change of samples was observed by TEM, and the activity of samples was measured as per the above enzymatic assays.

### 2.8. Stability of Free CAT, CAT/ZIF-8, and CAT/ZIF-8 with Nanocoating

The thermal stability of free CAT and immobilized CAT was investigated by incubating at 50 °C for 10–60 min, respectively. The sample was collected and assayed for enzyme activity. The pH stability of free CAT and immobilized CAT was measured in the system over a pH range between 3 and 11 for 1 h at 25 °C. The residual activities were determined. For the storage stability, free catalase, CAT/ZIF-8, SiO_2_@CAT/ZIF-8, and Fe^3+^-TA@CAT/ZIF-8 were stored at 25 °C. The residual activities of CAT samples were determined for a certain storage time. In addition, the reusability of immobilized CAT was evaluated by performing several consecutive operating cycles using 0.2% H_2_O_2_ solution as the substrate. The CAT/ZIF-8, SiO_2_@CAT/ZIF-8, and Fe^3+^-TA@CAT/ZIF-8 were collected and washed with 50 mM phosphate buffer (pH 7.5) solution after each batch and then added to the next cycle, respectively. The reusability was defined as the ratio of the activity for the immobilized CAT after recycling to its initial activity.

## 3. Results and Discussion

### 3.1. Synthesis and Characterization of the CAT@ZIF-8 Composites with a Protective Nanocoating

**Figure 2:**
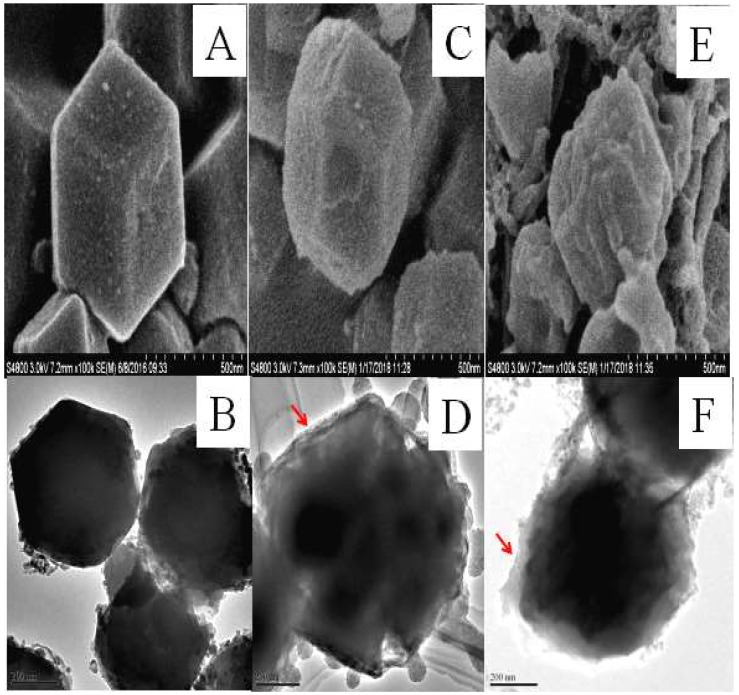
SEM images and TEM images of CAT/ZIF-8 (**A**,**B**), SiO_2_@CAT/ZIF-8 (**C**,**D**), and Fe^3+^-CA@CAT/ZIF-8 (**E**,**F**).

**Figure 3:**
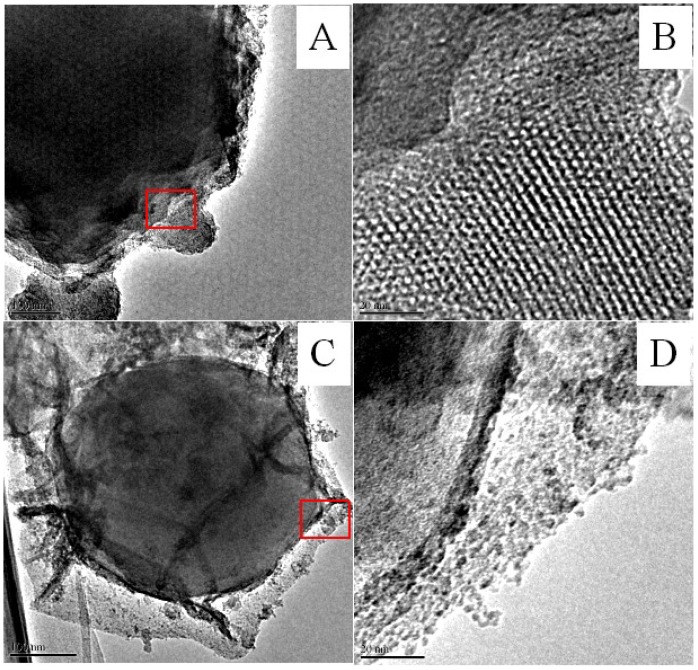
HRTEM images of SiO_2_@CAT/ZIF-8 (**A**,**B**) and Fe^3+^-CA@CAT/ZIF-8 (**C**,**D**).

**Figure 4:**
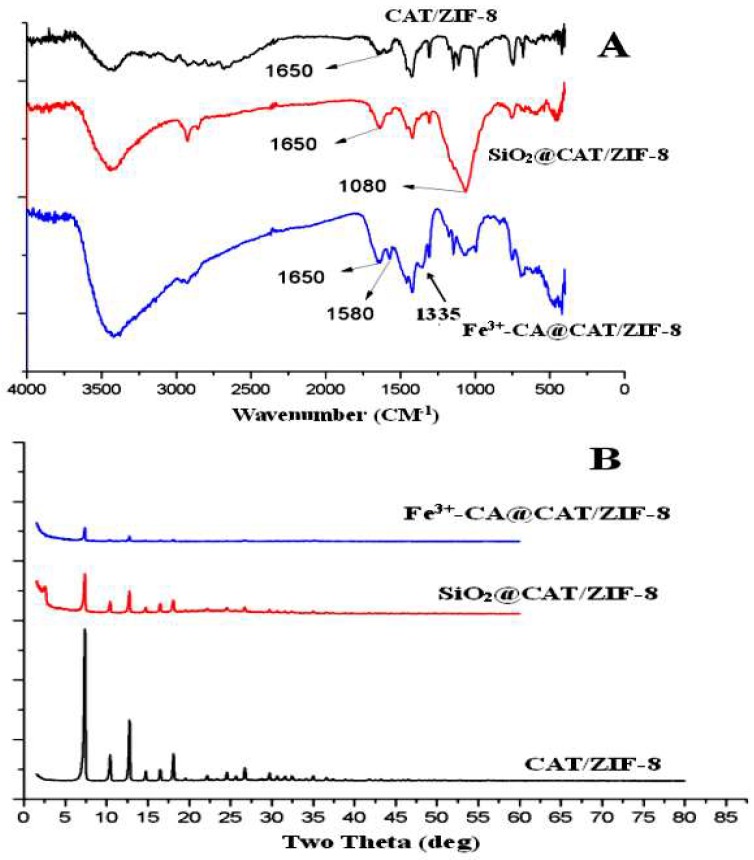
FTIR spectra (**A**) and powder X-ray diffraction (PXRD) pattern (**B**) of CAT/ZIF-8, SiO_2_@CAT/ZIF-8, and Fe^3+^-CA@CAT/ZIF-8.

**Figure 5:**
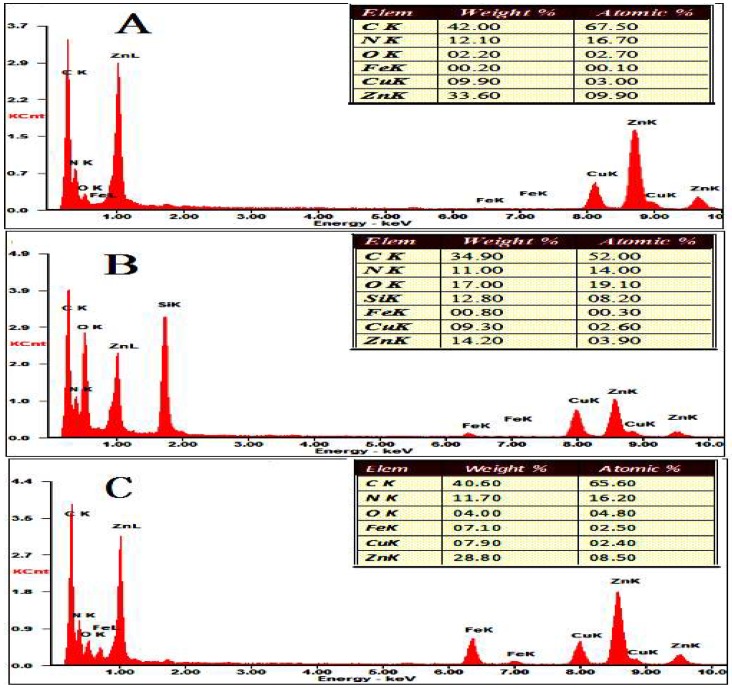
EDS patterns of CAT/ZIF-8 (**A**), SiO_2_2@CAT/ZIF-8 (**B**), and Fe^3+^-CA@CAT/ZIF-8 (**C**).

The synthesis of the CAT@ZIF-8 composites with a protective nanocoating are shown schematically in Figure 1, and the process comprises three steps. In the first step, ZIF-8 nanoparticles are synthesized by mixing 2-methylimidazole solution (0.05 M) and zinc nitrate solution (0.3 M) together and selected as carrier materials. Then, the CAT was adsorbed onto the ZIF-8 surface by mixing CAT solution and ZIF-8. In a final step, for the Fe^3+^-TA@CAT/ZIF-8 composites, the obtained CAT/ZIF-8 particles were mixed with TA and FeCl_3_·6H_2_O water solution to enable the growth of an Fe^3+^-TA nanocoating at the surface of the enzyme by the crystallization and deposition of Fe^3+^-TA crystals, thus resulting in the formation of a mesoporous Fe^3+^-TA nanocoating on the surface of the immobilized CAT. For SiO_2_@CAT/ZIF-8 composites, CTAB was utilized to direct the overgrowth of mesostructured silica on the external surface of CAT/ZIF-8. In this process, the self-assembled layer of CTAB molecules served as a structure-directing agent of the mesostructure, and it bridged the mesoporous silica and CAT/ZIF-8 as well. After centrifugation and washing, CAT@ZIF-8 composites with a protective nanocoating could be found by SEM (Figure 2). SEM images revealed that the CAT/ZIF-8 had a standard polyhedron morphology with relatively smooth surface (Figure 2A,C). Compared to CAT/ZIF-8, both SiO_2_@CAT/ZIF-8 and Fe^3+^-TA@CAT/ZIF-8 exhibited rough surfaces (Figure 2E). TEM images showed a thin and dense silica layer surrounding the CAT/ZIF-8, which was observed clearly for the SiO_2_@CAT/ZIF-8 (Figure 2B) compared to the CAT/ZIF-8 (Figure 2B,D). Likewise, a thick and loose Fe^3+^-TA film was formed around the CAT/ZIF-8 (Figure 2F). The nanocoating structure around CAT/ZIF-8 was further confirmed by a high resolution TEM image (Figure 3). Both the Fe^3+^-TA film and silica layer had a clear mesoporous structure (Figure 3B,D). In contrast to Fe^3+^-TA film, the silica layer exhibited an orientated mesoporous structure, and all the mesopores of silica were perpendicular to the surface of ZIF-8 cubes (Figure 3B). The FTIR spectra showed that the characteristic bands of CAT/ZIF-8, SiO_2_@CAT/ZIF-8 and Fe^3+^-TA@CAT/ZIF-8 at 1650 cm^−1^ were attributed to the amide I band, indicating the presence of CAT in the composites (Figure 4A) [38]. The band of SiO_2_@CAT/ZIF-8 at 1080 cm^−1^ was assigned to Si–O–Si [39]. However, the band was not observed in CAT/ZIF-8 and Fe^3+^-TA@CAT/ZIF-8. This result indicated the presence of SiO_2_ in the SiO_2_@CAT/ZIFs. Besides this, the bands at 1335 and 1580 cm^−1^ (catechol ring vibration) were observed, corresponding to the chemical structure of TA [40]. In addition, the PXRD pattern showed that the crystallinity of the SiO_2_@CAT/ZIF-8 and Fe^3+^-TA@CAT/ZIF-8 was unchanged compared to the experimental XRD patterns of CAT/ZIF-8 (Figure 4B), indicating that the incorporation of the silica layer or Fe^3+^-TA film did not significantly affect the morphology of the CAT/ZIF-8 crystals [41]. In addition, EDS analysis showed that the presence of the Fe element indicated that CAT was embedded in the CAT/ZIF-8, mSiO_2_@CAT/ZIF-8, and Fe^3+^-TA@CAT/ZIF-8 because CAT has four porphyrin heme (iron) groups. Meanwhile, the presence of the Zn and Si elements indicated the formation of ZIF-8 and a silica shell (Figure 5).

### 3.2. Acid Resistance Measurement of CAT/ZIF-8, SiO_2_@CAT/ZIF-8 and Fe^3+^-TA@CAT/ZIF-8

**Figure 6:**
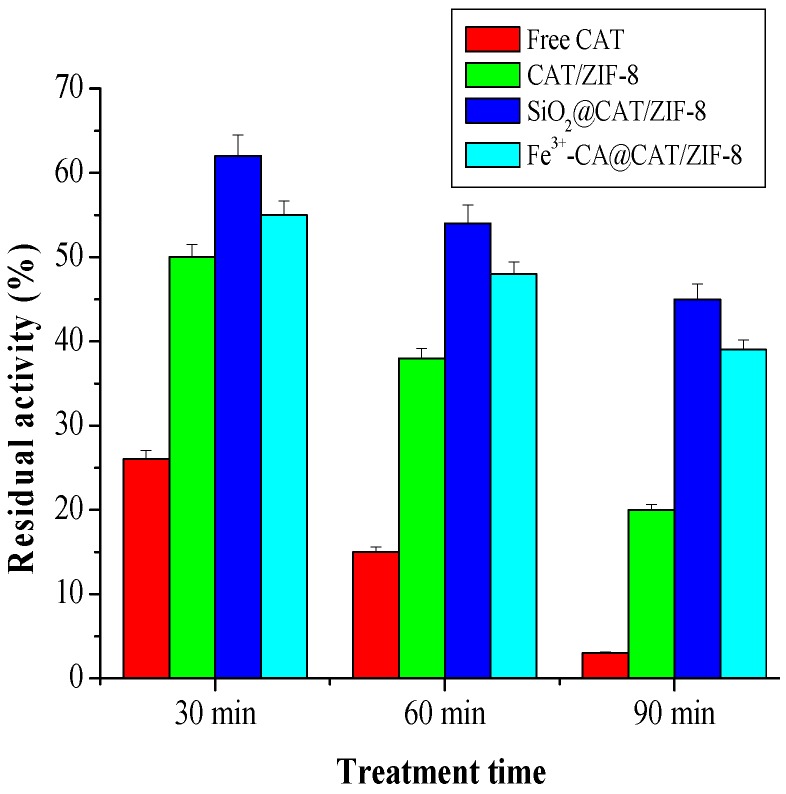
Acid tolerance (pH 3.0) of SiO_2_@CAT/ZIF-8 and Fe^3+^-CA@CAT/ZIF-8.

**Figure 7:**
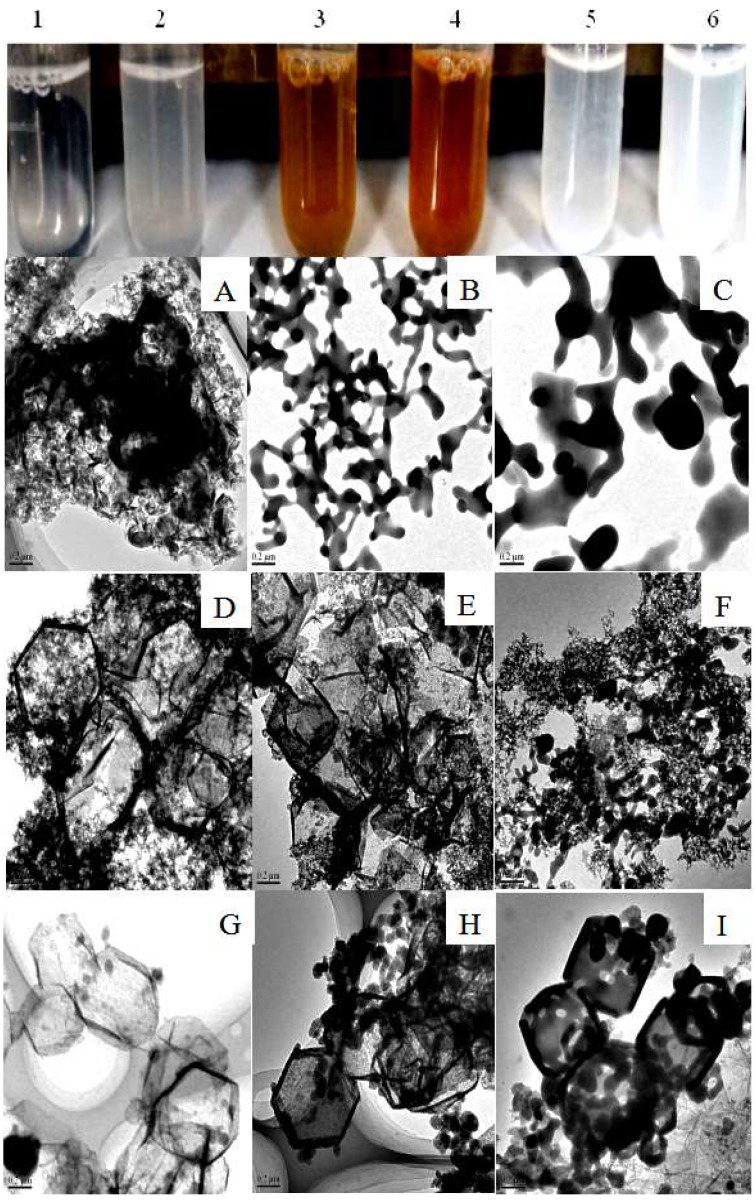
Appearance of CAT/ZIF-8 (1, after acid treatment; 2, before treatment), Fe^3+^-CA@CAT/ZIF-8 (3, after acid treatment; 4, before treatment), and SiO_2_@CAT/ZIF-8 (5, after acid treatment; 6, before treatment); and TEM images of CAT/ZIF-8 (**A**, **B**, and **C**, acid treatment for 30, 60, 90 min, respectively), Fe^3+^-CA@CAT/ZIF-8 (**D**, **E** and **F**, acid treatment for 30, 60, 90 min, respectively), and SiO_2_@CAT/ZIF-8 (**G**, **H** and **I**, acid treatment for 30, 60, 90 min, respectively).

**Figure 8:**
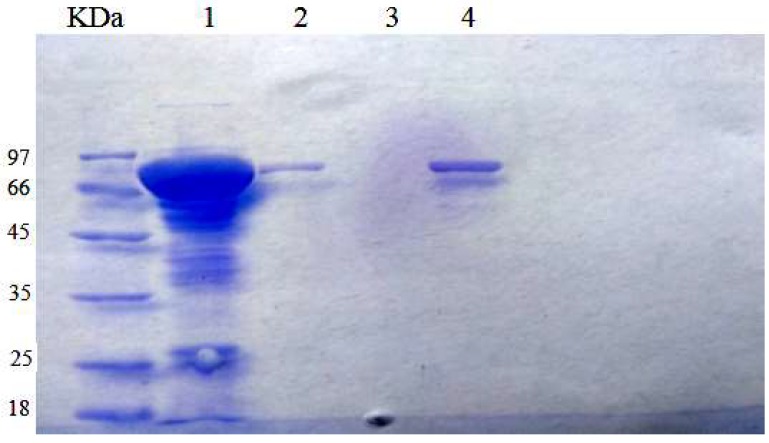
SDS-PAGE gel (M: protein marker, lane 1: free catalase, lane 2: CATZIF-8, lane 3: SiO_2_@CAT@ZIF-8, lane 4: Fe^3+^-TA@CAT/ZIF-8.

**Figure 9:**
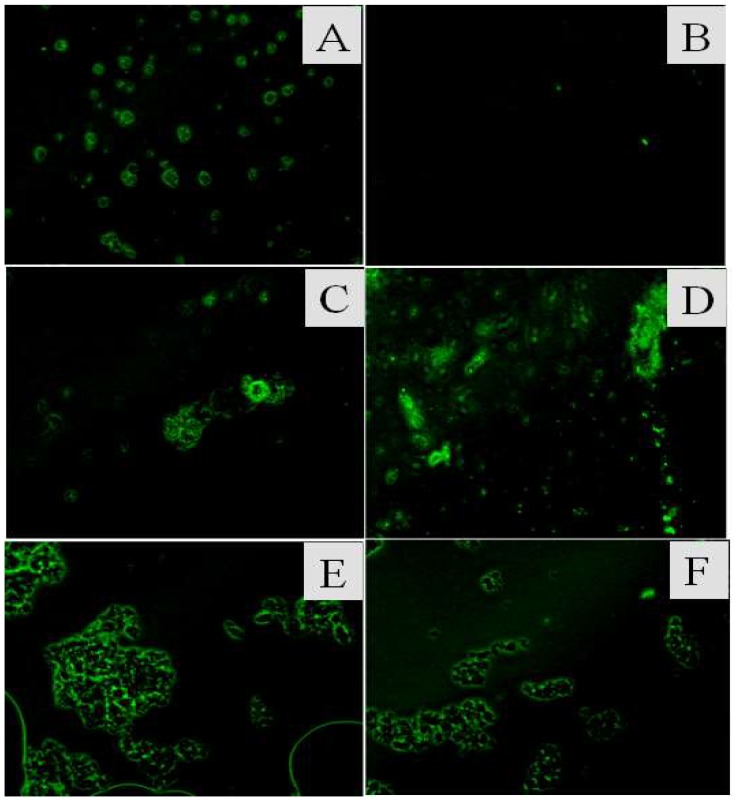
Confocal microscope images of CAT/ZIF-8, Fe^3+^-TA@CAT/ZIF-8, and SiO_2_@CAT@ZIF-8. (**A**,**C**,**E**) before acid treatment; (**B**,**D**,**F**) after acid treatment.

To assess whether the silica shell and Fe^3+^-TA film could improve the acid resistance of CAT/ZIF-8 composites, we examined the catalytic activity and pH stability of CAT/ZIF-8, SiO_2_@CAT/ZIF-8 and Fe^3+^-TA@CAT/ZIF-8 in an acidic environment (pH 3.0). The results are shown in Figure 6. Under an acidic environment, the activity of the three enzymes decreased. However, SiO_2_@CAT/ZIF-8 and Fe^3+^-TA@CAT/ZIF-8 showed more stable performance than CAT/ZIF-8 in the acidic environment (pH 3.0). CAT/ZIF-8 composites only maintained 20% of their initial activity (about 12 U/mg). However, SiO_2_@CAT/ZIF-8 and Fe^3+^-TA@CAT/ZIF-8 still retained 50% and 40% of their initial activity (about 10 U/mg), respectively. These results showed that the stability of CAT/ZIF-8 against acid degradation was remarkably improved due to the protection of nanocoating (silica layer or Fe^3+^-TA film) on the outside surface of CAT/ZIF-8. The increased tolerance against acid degradation may be due to the fact that the nanocoating can provide an appropriate microenvironment for the enzyme, and protect CAT from structural changes under extreme pH values [42]. Besides this, the turbidity of the three immobilized enzymes solutions exhibited obvious differences before and after acid treatment (Figure 7). After acid treatment, the three immobilized enzyme solutions became clearer than before acid treatment, indicating that all of the immobilized CAT experienced acid degradation. Furthermore, CAT/ZIF-8 solution became clearer than Fe^3+^-TA@CAT/ZIF-8 and SiO_2_@CAT/ZIF-8. At the same time, CAT/ZIF-8 solution after acid treatment displayed the minimum absorbance (OD_600_) compared with Fe^3+^-TA@CAT/ZIF-8 and SiO_2_@CAT/ZIF-8 (data not shown). The results showed that CAT/ZIF-8 was easily degraded by acid. TEM images showed that the morphologies of the three enzymes underwent changes under the acidic environment (pH 3.0). After acid treatment for 30 min, the complete polyhedron morphology could not be observed for CAT/ZIF-8 (Figure 7A). CAT/ZIF-8 composites experienced obvious degradation. With the increase of processing time, CAT/ZIF-8 composites were absolutely degraded, no intact CAT/ZIF-8 was observed, and only small nanoparticles are observed (Figure 7B,C). In contrast, Fe^3+^-TA@CAT/ZIF-8 retained their polyhedron morphology after acid treatment for 30 min (Figure 7D). After 60 min, the polyhedron morphology of Fe^3+^-TA@CAT/ZIF-8 could be clearly observed (Figure 7E). However, no intact Fe^3+^-TA@CAT/ZIF-8 was observed after 90 min, indicating that Fe^3+^-TA@CAT/ZIF-8 composites were degraded. It is worth noting that SiO_2_@CAT/ZIF-8 still retained its polyhedron morphology after acid treatment for 90 min (Figure 7G,H,I), which protected it from CAT leaching. Furthermore, the significant leaching of CAT in CAT/ZIF-8 under an acid environment was further confirmed by SDS-PAGE. CAT/ZIF-8, Fe^3+^-TA@CAT/ZIF-8 and mSiO_2_@CAT/ZIFs were dissolved under an acid environment (pH 3.0) for 90 min and the CAT protein molecules were released. SDS-PAGE was then used to determine the presence of CAT protein molecules (Figure 8). Protein bands corresponding to the molecular weight of CAT (the subunit of CAT) appeared on the gel for free CAT, CAT/ZIF-8 and Fe^3+^-TA@CAT/ZIF-8 samples (lane 1, lane 2 and lane 4). However, no band was observed for the SiO_2_@CAT/ZIFs sample (lane 3). These results indicated that the embedded protein molecules in CAT@ZIF-8 were easily released from ZIF-8 scaffolds once digested by acetic acid, whereas the embedded protein molecules in CAT/ZIF-8 with a protective nanocoating were difficult to released due to the protection of the nanocoating for CAT protein. This hypothesis was further confirmed by using CLSM. The CLSM images showed that the fluorescently labeled CAT molecules were clearly present in the CAT/ZIF-8, Fe^3+^-TA@CAT/ZIF-8 and SiO_2_@CAT/ZIF-8 before acid treatment, suggesting that CAT molecules were embedded in the CAT/ZIF-8, Fe^3+^-TA@CAT/ZIF-8 and SiO_2_@CAT/ZIF-8, respectively (Figure 9A,C,E). However, after acid treatment for 60 min, no fluorescence was observed for CAT/ZIF-8, indicating that the embedded CAT molecules in CAT@ZIF-8 were released from the ZIF-8 scaffolds due to acid degradation (Figure 9B). In contrast, the fluorescently labeled CAT molecules were clearly observed in the Fe^3+^-TA@CAT/ZIF-8 and SiO_2_@CAT/ZIF-8 (Figure 9D,F). These results showed that the stability of CAT/ZIF-8 against acid degradation was remarkably improved due to the protection of nanocoating on the outside surface of CAT/ZIF-8.

### 3.3. Stability of CAT/ZIF-8, Fe^3+^-TA@CAT/ZIF-8 and SiO_2_@CAT/ZIF-8

**Figure 10:**
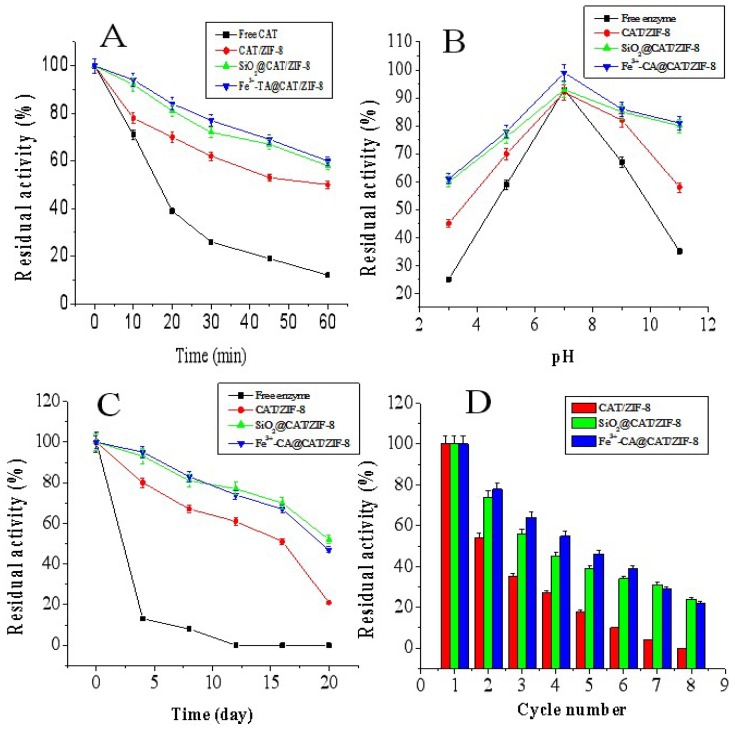
Stability of free CAT, CAT/ZIF-8, Fe^3+^-TA@CAT/ZIF-8, and SiO_2_@CAT@ZIF-8. (**A**) thermostability, (**B**) pH-stability, (**C**) storage stability, (**D**) reusability.

Generally, immobilization can improve the stability of enzymes in extreme conditions such as high temperatures [43,44]. We examined the stability of the free CAT, CAT/ZIF-8, Fe^3+^-TA@CAT/ZIF-8, and SiO_2_@CAT/ZIF-8 against heat. The results are shown in Figure 10A. Immobilized CAT showed a more stable performance than free CAT at 50 °C. Compared to CAT/ZIF-8, Fe^3+^-TA@CAT/ZIF-8, and SiO_2_@CAT/ZIF-8 exhibited better thermal stability. The increased tolerance towards high temperatures may be due to the fact that the nanocoating/enzyme interactions reduced the conformational changes of CAT, preventing enzyme dissociation. Likewise, Fe^3+^-TA@CAT/ZIF-8, and SiO_2_@CAT/ZIF-8 also displayed more stability than free CAT and CAT/ZIF-8 against extreme pH values. For example, Fe^3+^-TA@CAT/ZIF-8 and SiO_2_@CAT/ZIF-8 still retained about 80% of their initial activity (about 10 U/mg) at pH 11 for 1 h, respectively. However, free CAT and CAT/ZIF-8 (about 12 U/mg) only retained 35% and 60% of their initial activity, respectively (Figure 10B). In addition, the storage stability of free CAT and immobilized CAT was also evaluated. As shown in Figure 10C, the SiO_2_@CAT/ZIF-8 and Fe^3+^-TA@CAT/ZIF-8 could retain 50% of their initial activity after 20 days. In contrast, free CAT almost lost all of its activity, while the CAT/ZIF-8 only maintained 20% of initial activity. It was likely that the nanocoatings provided a hydrophilic microenvironment for the enzyme, thus reducing the possible distortion effect on the active structure of the enzyme. Besides this, the reusability of CAT/ZIF-8, Fe^3+^-TA@CAT/ZIF-8, and SiO_2_@CAT/ZIF-8 for performing several consecutive operating cycles using 0.2% H_2_O_2_ solution as the substrate was evaluated. As shown in Figure 10D, the SiO_2_@CAT/ZIF-8 and Fe^3+^-TA@CAT/ZIF-8 still retained about 40% of their initial activity after the 7 cycles, whereas CAT/ZIF-8 lost activity, suggesting that the CAT/ZIF-8 with a protective nanocoating has better reusability than CAT/ZIF-8. The suitable stability enabled the immobilized enzyme to be reused in industrial applications.

## 4. Conclusions

In conclusion, we successfully synthesized novel CAT@ZIF-8 composites with protective nanocoatings. The CAT@ZIF-8 composites with protective nanocoatings exhibited better thermostability, storability and reusability than conventional CAT/ZIF-8 composites. More importantly, the acid resistance of CAT/ZIF-8 was significantly improved due to the presence of nanocoatings. Thus, we have provided an efficient strategy for preparing stable enzyme/ZIF-8 composites with acid resistance, which has potential for practical applications even in acidic environments.

## References

[1] Dicosimo R., McAuliffe J., Poulose A.J., Bohlmann G. (2013). Industrial use of immobilized enzymes. Chem. Soc. Rev..

[2] Cui J.D., Jia S.R. (2015). Optimization protocols and improved strategies of cross-linked enzyme aggregates technology: Current development and future challenges. Crit. Rev. Biotechnol..

[3] Franssen M.C.R., Steunenberg P., Scott E.L., Zuilhof H., MSanders J.P. (2013). Immobilised enzymes in biorenewables production. Chem. Soc. Rev..

[4] Feng W., Ji P.J. (2011). Enzymes Immobilized on carbon nanotubes. Biotechnol. Adv..

[5] Barbosa O., Torrest R., Ortiz C., Berenguer-Murcia A., Rodrigues R.C., Fernandez-Lafuente R. (2013). Heterofunctional supports in enzyme immobilization: From traditional immobilization protocols to ppportunities in tuning enzyme properties. Biomacromolecules.

[6] Cao L.Q. (2005). Immobilised enzymes: Science or art?. Curr. Opin. Chem. Biol..

[7] Jesionowski T., Zdarta J., Krajewska B. (2014). Enzyme immobilization by adsorption: A review. Adsorption.

[8] Carcia-Galan C., Berenguer-Murcia A., Fernandez-Lafuente R., Rodrigues R.C. (2011). Potential of different enzyme immobilization strategies to improve enzyme performance. Adv. Synth. Catal..

[9] Quilles J.C.J., Brito R.R., Borges J.P.C., Aragon C., Fernandez-Lorente G., Bocchini-Martins D.A., Gomes E., Da Silva R., Boscolo M., Guisan J.M. (2015). Modulation of the activity and selectivity of the immobilized lipases by surfactants and solvents. Biochem. Eng. J..

[10] Chen Q., Kenausis G.L., Heller A. (1998). stability of oxidases immobilized in silica gels. J. Am. Chem. Soc..

[11] Wang Q.G., Yang Z.M., Zhang X.Q., Xiao X.D., Chang C.K., Xu B.A. (2007). Supramolecular-hydrogel-encapsulated hemin as an artificial enzyme to mimic peroxidase. Angew. Chem. Int. Ed..

[12] Ansari S.A., Husain Q. (2012). Potential applications of enzymes immobilized on/in nano materials: A review. Biotechnol. Adv..

[13] Furukawa H., Cordova K.E., O’Keeffe M., Yaghi O.M. (2013). The chemistry and applications of metal-organic frameworks. Science.

[14] Mateo C., Palomo J.M., Fernandez-Lorente G., Guisan J.M., Fernandez-Lafuente R. (2007). Improvement of enzyme activity, stability and selectivity via immobilization techniques. Enzyme Microb. Technol..

[15] Hudson S., Cooney J., Magner E. (2008). Proteins in mesoporous silicates. Angew. Chem. Int. Ed. Engl..

[16] Nadar S.S., Rathod V.K. (2018). Encapsulation of lipase within metal-organic framework (MOF) with enhanced activity intensified under ultrasound. Enzymes Microb. Technol..

[17] Kempahanumakkagari S., Kumar V., Samaddar P., Kumar P., Ramakrishnappa T., Kim K.H. (2018). Biomolecule-embedded metal-organic frameworks as an innovative sensing platform. Biotechnol. Adv..

[18] Furukawa H., Ko N., Go Y.B., Aratani N., Choi S.B., Choi E., Yazaydin A.O., Snurr R.Q., OKeeffe M., Kim J. (2010). Ultrahigh porosity in metal-organic frameworks. Science.

[19] Li P., Moon S.Y., Guelta M.A., Harvey S.P., Hupp J.T., Farha O.K. (2016). Encapsulation of a nerve agent detoxifying enzyme by a mesoporous zirconium metal–organic framework engenders thermal and long-term stability. J. Am. Chem. Soc..

[20] Feng D.W., Liu T.F., Su J., Bosch M., Wei W., Yuan D.Q., Chen Y.P., Wang X.K., Wang C., Lian X.Z. (2015). Stable metal-organic frameworks containing single-molecule traps for enzyme encapsulation. Nat. Commun..

[21] Li P., Kelt R.C., Moon S.Y., Wang T.C., Deria P., Peters A.W., Kalhr B.M., Park H.J., Al-Juaid S.S., Hupp J.T. (2015). Synthesis of nanocrystals of Zr-based metal–organic frameworks with Csq-Net: Significant enhancement in the degradation of a nerve agent simulant. Chem. Commun..

[22] Gkaniatsou E., Sicard C., Ricoux R., Mahy J.P., Steunou N., Serre C. (2017). Metal–organic frameworks: A novel host platform for enzymatic catalysis and detection. Mater. Horiz..

[23] Lian X.Z., Fang Y., Joseph E., Wang Q., Li J.L., Banerjee S., Lallar C., Wang X., Zhou H.C. (2017). Enzyme-MOF (metal–organic framework) composites. Chem. Soc. Rev..

[24] Wu Y., Zhou M., Zhang B., Wu B., Li J., Qiao J., Guan X., Li F. (2014). Amino acid assisted templating synthesis of hierarchical zeolitic imidazolate framework-8 for efficient arsenate removal. Nanoscale.

[25] Wu X.L., Yang C., Ge J., Liu Z. (2015). Polydopamine tethered enzyme/metal–organic framework composites with high stability and reusability. Nanoscale.

[26] Park K.S., Côté Z.N., Choi A.P., Huang J.Y., Uribe-Romo R., Chae F.J., ÓKeeffe H., Yaghi O.M. (2006). Exceptional chemical and thermal stability of zeolitic imidazolate frameworks. Proc. Natl. Acad. Sci. USA.

[27] Banerjee R., Furukawa H., Britt D., Knobler C., O’Keeffe M., Yaghi O.M. (2009). Control of pore size and functionality in isoreticular zeolitic imidazolate frameworks and their carbon dioxide selective capture properties. J. Am. Chem. Soc..

[28] Falcaro P., Ricco R., Doherty C.M., Liang K., Hill A.J., Styles M.J. (2014). MOF positioning technology and device fabrication. Chem. Soc. Rev..

[29] Zou Z., Li S., He D., He X., Wang K., Li L., Yang X., Li H. (2017). A versatile stimulus-responsive metal–organic framework for size/morphology tunable hollow mesoporous silica and pH-triggered drug delivery. J. Mater. Chem. B.

[30] Hidalgo A., Betancor L., Lopez-Gallego F., Moreno R., Berenguer J., Fernández-Lafuente R., Guisan R.J.M. (2003). Design of an immobilized preparation of catalase from *Thermus thermophilus* to be used in a wide range of conditions.: Structural stabilization of a multimeric enzyme. Enzyme Microb. Technol..

[31] Hernandez K., Berenguer-Murcia A., Rodrigues R.C., Fernandez-Lafuente R. (2012). Hydrogen peroxide in biocatalysis. A dangerous liaison. Curr. Org. Chem..

[32] Betancor L., Hidalgo A., Fernandez-Lorente G., Mateo C., Fernandez-Lafuente R., Guisan J.M. (2003). Preparation of a stable biocatalyst of bovine liver catalase using immobilization and postimmobilization techniques. Biotechnol. Prog..

[33] Kauldhar B.S., Dhau J.S., Sooch B.S. (2016). Covalent linkage of alkalothermophilic catalase onto functionalized cellulose. RSC Adv..

[34] Li J., Li L.S., Xu L. (2017). Hierarchically macro/mesoporous silica spheres for catalase immobilization and catalysis. Mater. Lett..

[35] Shieh F.K., Wang S.C., Yen C.I., Wu C.C., Dutta S., Chou L.Y., Morabito J.V., Hu P., Hsu M.H., Wu K.C.W. (2015). Imparting functionality to biocatalysts via embedding enzymes into nanoporous materials by a de Novo approach: Size-selective sheltering of catalase in metal-organic framework microcrystals. J. Am. Chem. Soc..

[36] Cui J., Feng Y., Lin T., Tan Z., Zhong C., Jia S. (2017). Mesoporous metal–organic framework with well-defined cruciate flower-like morphology for enzyme immobilization. ACS Appl. Mater. Interfaces.

[37] Seyhan Tukel S., Hurrem F., Yildirim D., Alptekin O. (2013). Preparation of crosslinked enzyme aggregates (CLEA) of catalase and its characterization. J. Mol. Catal. B Enzym..

[38] Gao J., Kong W.X., Zhou L., He Y.Y., Ma L., Wang Y., Yin L.Y., Jiang Y.J. (2017). Monodisperse core-shell magnetic organosilica nanoflowers with radial wrinkle for lipase immobilization. Chem. Eng. J..

[39] Kato K., Kawachi Y., Nakamura H. (2014). Silica–enzyme–ionic liquid composites for improved enzymatic activity. J. Asian Ceram. Soc..

[40] Park J.H., Choi S.H., Moon H.C., Seo H.L., Kim J.Y., Hong S.P., Lee B.S., Kang E., Lee J.H., Ryu D.H. (2017). Antimicrobial spray nanocoating of supramolecular Fe(III)-tannic acid metal-organic coordination complex: Applications to shoe insoles and fruits. Sci. Rep..

[41] Cui J.D., Ren S.Z., Lin T., Feng Y.X., Jia S.R. (2018). Shielding effects of Fe^3+^-tannic acid Films for immobilized enzyme on magnetic Fe_3_O_4_@silica core shell nanosphere. Chem. Eng. J..

[42] Shi J.F., Tian Y., Liu H., Yang D., Zhang S.H., Wu Y.H., Jiang Z.Y. (2017). Shielding of enzyme by stable and protective organosilica layer on monolithic scaffolds for continuous bioconversion. Ind. Eng. Chem. Res..

[43] Fernandez-Lafuente R. (2009). Stabilization of multimeric enzymes: Strategies to prevent subunit dissociation. Enzyme Microb. Technol..

[44] Zhou Z., Hartmann M. (2013). Progress in enzyme immobilization in ordered mesoporous materials and related applications. Chem. Soc. Rev..

